# Colitis due to cancer treatment with immune check-point inhibitors – review of literature and presentation of clinical cases

**DOI:** 10.2478/raon-2024-0022

**Published:** 2024-03-22

**Authors:** Andreja Ocepek

**Affiliations:** Department of Gastroenterology, Division of Internal Medicine, University Medical Centre Maribor, Maribor, Slovenia

**Keywords:** immune checkpoint inhibitors, immune checkpoint inhibitors induced colitis, immune-mediated microscopic colitis, corticosteroid therapy, infliximab, vedolizumab

## Abstract

Treatment with immune checkpoint inhibitors is effective in various cancers, but may be associated with immune-mediated side effects in other organs. Among the more common ones is gastrointestinal tract involvement, especially colitis. In most patients, colitis is mild or responds to corticosteroid treatment. A smaller proportion of patients, more often those treated with cytotoxic T lymphocyte antigen-4 inhibitors, may have a more severe course of colitis, even life-threatening complications. In these patients, prompt action, timely diagnosis with endoscopic evaluation and early treatment with high-dose corticosteroids and, if ineffective, rescue therapy with biologic agents such as infliximab and vedolizumab are needed. We present three cases from our clinical practice, data on incidence and clinical presentation, current recommendations regarding diagnostic approach and treatment of immune checkpoint inhibitors induced colitis.

## Introduction

Inhibition of immune checkpoints strengthens the body's own defences against cancer, which can be exploited to great advantage in the treatment of malignant diseases. Such drugs are antibodies against inhibitory immune regulators such as cytotoxic T lymphocyte antigen-4 (CTLA-4), programmed cell death protein-1 (PD-1) and programmed death protein-1 ligand (PD-L1). Due to their involvement in the immune response, immune checkpoint inhibitors (ICPI) can trigger other immune-mediated diseases as a side effect, affecting many other organs and tissues, *e.g.* skin, lung, liver and gastrointestinal tract. The most common side effect of immunotherapy for cancer of the gastrointestinal tract is immune-mediated or ICPI-induced colitis.^1,2^

## Incidence

The incidence of ICPI-induced colitis depends on three factors: the type of immunotherapy, the characteristics of the patient and the characteristics of the cancer. Colitis occurs more frequently with CTLA-4 inhibitors than with PD-1 and PD-L1 inhibitors, with combination therapy with two ICPIs, and with higher doses, although a clear dose-dependence of the occurrence and severity of colitis has not been confirmed.^3^ The incidence of colitis is 3.4–22% with CTLA-4 inhibitor therapy, 0.7–12.8% with combined CTLA-4 and PD-1 inhibitor therapy, and 0.7–2.6% with PD-1/PD-L1 inhibitor monotherapy.^1,2^ Another important factor is cancer itself. According to data known so far, the incidence of colitis is higher in patients with melanoma than in patients with other cancers. For example, ipilimumab treatment causes diarrhoea in 41% of melanoma patients compared with up to 27% of lung cancer patients.^3^ The stage of cancer may also influence the incidence of side-effects, although the underlying mechanism is unknown. Thus, the incidence of diarrhoea and colitis is lower in stage IV (35.3%) than in stage III (72%) of the same cancer.^1^ Third factor is the patient himself. The risk of ICPI-induced colitis is higher in Caucasians and in patients with known inflammatory bowel disease (IBD), but the influence of sex, age, gut microbiota and possibly genetic predisposition (*e.g.* human leukocyte antigen (HLA) status) remains unclear.^1,3^

## Clinical presentation

Symptoms and signs of ICPI-induced colitis most commonly include diarrhoea, abdominal pain, bloating, haemochesia, mucus discharge, fever and vomiting.^1,3^ Diarrhoea occurs in most cases 4–8 weeks after the start of ICPI, but in some patients it may be as early as 1 week, and in others it may occur months or up to 2 years after the end of treatment.^1^ The severity of colitis may range from mild, self-limiting diarrhoea to life-threatening colitis with the risk of rapid onset of complications such as ileus, toxic megacolon and intestinal perforation.^1,2^ The severity of diarrhoea and colitis is defined by the Common Terminology Criteria for Adverse Events (CTCAE) in 5 grades shown in Table 1.^2,4,5^ A more severe course of colitis can be expected in patients receiving CTLA-4 inhibitors (*e.g.* ipilimumab) and in patients with known IBD.^2,3^

**TABLE 1.** CTCAE gastrointestinal toxicity levels of ICPI^2,4^

**Grade**	**Diarrhoea**	**Colitis**
1	Increase in frequency of bowel movements < 4x above baseline; mild increase in ejection via stoma	Asymptomatic; clinical or diagnostic observation only; no action required
2	Increase in frequency of bowel movements 4–6x above baseline; moderate increase in ejection via stoma	Abdominal pain; mucus or blood in stool
3	Increase in frequency of bowel movements ≥ 7x above baseline, incontinence, need for hospitalisation, impaired daily self-care; marked increase in ejection via stoma	Severe or persistent abdominal pain; change in bowel movements; fever, ileus, peritoneal irritation; medical intervention required
4	Life-threatening consequences; need for urgent action	Life-threatening consequences; need for urgent action
5	Death	Death

CTCAE = Common Terminology Criteria for Adverse Events; ICPI = immune checkpoint inhibitors

## Diagnosis

ICPI-induced colitis should be considered in all cancer patients receiving ICPI. Because of non-specific symptoms, diagnosis is based on exclusion of other conditions with a similar clinical presentation. In the differential diagnosis, we must consider infectious causes (*e.g.* clostridial diarrhoea, cytomegalovirus reactivation, *etc.*), drug-induced colitis (*e.g.* non-steroidal antirheumatic drugs, chemotherapeutic drugs, mycophenolate mofetil...), microscopic colitis, IBD flare, diverticulitis, ischaemic colitis, graft-versus-host disease, ICPI-induced pancreatitis, exocrine pancreatic insufficiency, ICPI-induced coeliac disease, thyroid dysfunction^1,4,6,7^. The recommended set of diagnostic tests and procedures is shown in Table 2.^1,2,3,8^ In case of severe abdominal pain or suspected complications, imaging is required, most commonly with computed tomography (CT) and/or magnetic resonance imaging (MRI). Both CT and MRI are insufficiently sensitive and specific for the diagnosis of ICPI-induced colitis.^2,3,4^

**TABLE 2.** Set of diagnostic tests^1,2,3^

**Stool tests**

Stool culture for pathogenic bacteria (*e.g.* Yersinia) and viruses
Clostridium difficile toxin
Parasites and parasite eggs
Pancreatic elastase
Inflammatory markers (calprotectin, lactoferrin)
PCR for CMV

**Blood tests**

Complete blood count
Biochemical tests (liver tests, renal function tests, amylase, lipase, albumin,...)
TSH
Inflammatory markers (CRP, ESR)
Serology for celiac disease (total IgA, tTG)
HIV serology

**Imaging**

CT scan of the abdomen (for severe abdominal pain)
Endoscopy of the lower GI tract (in all or at least patients with CTCAE grade ≥ 2)

**Investigations before immunosuppressive/biological treatment**

Serology for HAV, HBV and HCV, HIV
Quantiferon test
Chest X-ray

CMV = cytomegalovirus; CRP = C-reactive protein; CT = computed tomography; CTCAE = Common Terminology Criteria for Adverse Events; ESR = erythrocyte sedimentation rate; GI = gastrointestinal; HAV = hepatitis A virus; HBV = hepatitis B virus; HCV = hepatitis C virus; HIV = human immunodeficiency virus; IgA = immunoglobulin A; PCR = polymerase chain reaction; TSH = thyroid-stimulating hormone; tTG = tissue transglutaminase

## Endoscopic and histopathological features

Endoscopic findings are non-specific. Up to 37% of patients with ICPI-induced colitis have normal endoscopic findings, but up to 90% of patients with grade 1 colitis have microscopic changes, so even macroscopically normal mucosa needs to be biopsied for histological analysis. Endoscopically, mucosal oedema, erythema, erosions, loss of vascular permeation, superficial or deep ulcers may be found.^2^ Lesions may be present diffusely, segmentally or irregularly along the bowel. Single atypical cases of colonic pseudolipomatosis and collagenous colitis after atezolizumab treatment have also been described.^9,10^ The Mayo endoscopic scoring system, which is routinely used to describe ulcerative colitis, can be used to describe endoscopic changes (Table 3).^4,11^

**TABLE 3.** Mayo endoscopic score^14^

	**Mayo score 0**	**Mayo score 1**	**Mayo score 2**	**Mayo score 3**
Disease activity	Inactive disease	Mild activity erythema, decreased vascular pattern, mild friability	Medium activity marked erythema, absent vascular pattern, friability, erosions	High activity
Features	normal mucosa			spontaneous bleeding, ulceration

Clinical studies have shown the importance of endoscopic diagnosis in patients with CTCAE grade > 1 in predicting the need for biologic therapy, as patients with a more severe endoscopic picture according to Mayo endoscopic score of grade 3 were statistically significantly more likely to require treatment with infliximab.^12,13^

Histo-pathological features vary from acute colitis with intraepithelial neutrophilic infiltration, cryptitis and crypt abscesses, to chronic colitis with basal lymphocytic infiltration, Paneth cell metaplasia and disrupted crypt architecture, or both.^3,10^ Histo-pathological assessment of the severity of inflammation using the Nancy score has also been shown to be a good predictor of the need for biologic therapy, with 50% of patients with Nancy grade 3 and 4 requiring salvage therapy with infliximab compared with 20% of patients with Nancy grade 1 and 2.^1^

## Immune-mediated microscopic colitis

Immune-mediated microscopic colitis, recognised as a separate disease, presents with chronic watery diarrhoea. It usually occurs after treatment with anti-PD-1 or anti-CTLA-4. On colonoscopy, the intestinal mucosa is normal or mildly altered with oedema and/or erythema. Histopathological examination is the key to the diagnosis, and therefore random biopsies of apparently normal mucosa should always be performed. There are two forms of microscopic colitis. Lymphocytic colitis is characterised by intraepithelial lymphocytosis and infiltration of the lamina propria. In collagenous colitis, however, thickening of the collagenous subepithelial layer is visible on histopathology.^16^

## Treatment

Treatment depends on the stage of CTCAE. In grade 1, treatment is supportive and symptomatic. Oral hydration, a soft, non-irritating diet without lactose and caffeine, and antidiarrhoic drugs (*e.g.* loperamide) are advised after exclusion of infection.^15,16^ Treatment with mesalazine may be attempted.^3,17^ Discontinuation of ICPI is not necessary. If symptoms do not resolve within 7–10 days or the clinical picture worsens to grade ≥ 2, a consultation with a gastroenterologist and an endoscopic examination is recommended as a starting point for further treatment.^2,4,8^ In the absence of blood in the stool and/or normal endoscopic findings (Mayo grade 0), especially in confirmed microscopic colitis treatment with the topically acting corticosteroid, budesonide (extended-release tablets) at a dose of 9 mg daily may be attempted.^4,8,16^ Duration of treatment depends on whether ICPI is discontinued or not. If ICPI is discontinued, budesonide treatment lasts for 8 weeks, but if ICPI is continued, budesonide treatment can be maintained continuously. In the event of a discontinuation of budesonide, a gradual dose reduction from 9 mg to 6 mg for 14 days and then 3 mg for 14 days is advised, with careful monitoring of symptoms and, if necessary, extending time intervals.^4,16^ Mayo grade 1–2 colitis is treated primarily with a systemic corticosteroid. Patients without systemic signs of food-borne inflammation are prescribed the equivalent of prednisone 0.5–1 mg/kg/day, the dose of which is tapered (by 10 mg every 5–7 days) until discontinuation after symptoms subside. In case of systemic involvement, hospitalisation is usually necessary and intravenous methylprednisolone 0,5–1 mg/kg/day is recommended in a divided dose every 12 hours. With long-term corticosteroid treatment, prophylaxis of pneumocystis infection, vitamin D and calcium replacement and blood sugar control are recommended.^4,16^ The most severe endoscopic Mayo grade 3 requires treatment with higher doses of methylprednisolone 1–2 mg/kg/day intravenously and early consideration of salvage biologic therapy. In approximately 2/3 of patients, corticosteroid therapy is sufficient and is gradually weaned over 4–8 weeks.^1,8,16^ However, if the colitis does not resolve with corticosteroid treatment within 2–5 days, treatment with one of the biologic agents, TNF-a inhibitor infliximab or integrin a4b7 inhibitor vedolizumab, is indicated.^2,6^ The recommended dose of infliximab is 5–10 mg/kg infused at weeks 0, 2 and 6, and vedolizumab dose is 300 mg infused following the same regime.^1,3,4^ Treatment can be continued with a maintenance dose every 8 weeks if needed, depending on endoscopic reassessment after 8–10 weeks and decision to continue treatment with ICPI.^2^ In all patients with colitis grade ≥ 2, at least temporary discontinuation of ICPI and consideration of permanent discontinuation of CTLA-4 inhibitor or substitution with PD-1/PD-L1 inhibitor is necessary.^3^ In rare cases, all treatments described so far are ineffective and refractory colitis is present. In such cases, infectious causes should be excluded again, and alternative treatments such as other immunosuppressive drugs (*e.g.* mycophenolate mofetil, tacrolimus, cyclosporine), faecal transplantation or extracorporeal photopheresis are possible.^1,3,4^ Cases of treatment of refractory colitis with ustekinumab, tofacitinib and abatacept have also been described, but caution is advised due to the involvement of these drugs in anti-tumour response.^2^

## Prognosis

Overall mortality from ICPI-induced colitis is 5%. A more severe disease course should be considered in patients treated with a CTLA-4 inhibitor or with dual ICPI therapy, as the disease can be progressive and rapidly leads to life-threatening complications within 14 days.^1^

In most cases, recurrence of ICPI-induced colitis after treatment with corticosteroids or biologics does not occur if ICPI treatment is discontinued. The decision to restart ICPI treatment depends on the nature of cancer, severity and likelihood of recurrence of colitis, and availability of alternative treatments. After mild grade 1 colitis has subsided, continuation with the same ICPI is advised. Likelihood of recurrence of colitis is higher with CTLA-4 inhibitor therapy or dual (CTLA-4 inhibitor + PD-1/PD-L1 inhibitor) therapy, and therefore CTLA-4 inhibitor therapy is advised against after severe colitis has subsided. In this case, a switch to a PD-1/PD-L1 inhibitor in monotherapy is possible, and concomitant maintenance therapy with a biologic may also be considered. At present, evidence for long-term safety of such combination therapy is limited.^2,4^

## Clinical cases

### Case 1

**FIGURE 1.**
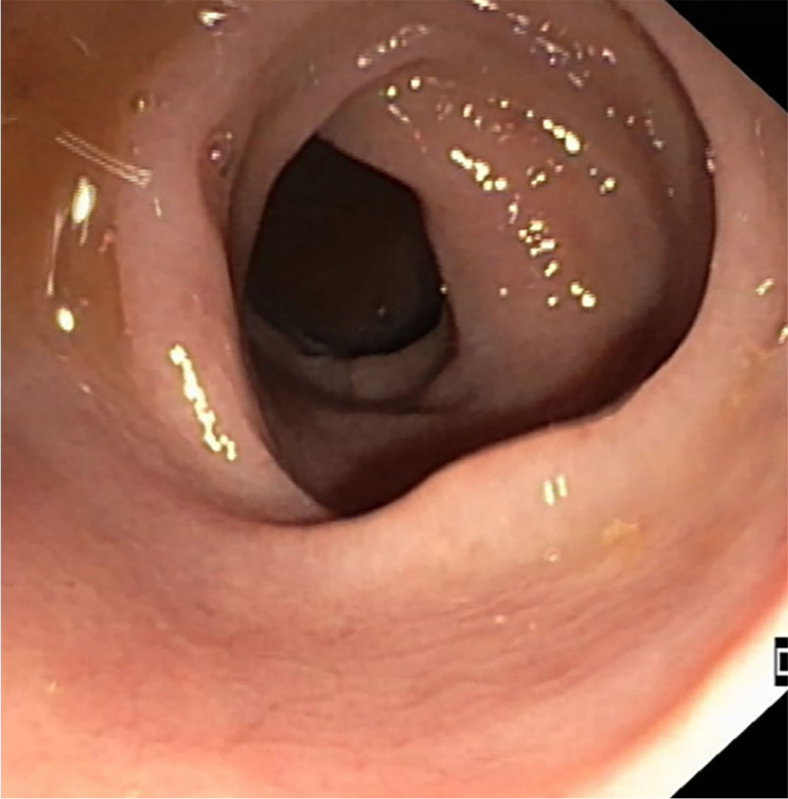
Endoscopic image of sigmoid colon in case 1.

A 71-year-old man with metastatic non-small cell K-ras positive lung cancer was treated with the PD-1 inhibitor pembrolizumab as a second-line oncologic therapy. After the 3^rd^ dose of immunotherapy, diarrhoea occurred, grade 2. CRP was 5 mg/L or less, faecal calprotectin was not determined. After excluding an infectious cause, he was treated with loperamide, followed by oral mesalazine and methylprednisolone at a dose of 0.5 mg/kg/day orally. Colonoscopy showed mild hyperaemia and oedema of the sigmoid mucosa, endoscopic Mayo score 1 (Figure 1), with normal mucosa in the rest of the colon.

Random biopsies of endoscopically normal mucosa and biopsies of sigmoid mucosa were histo-pathologically defined as moderate-to-intense aetiologically undefined chronic colitis with predominant plasma-cell mixed-cell inflammatory infiltrate. Following the decision of the multidisciplinary team, the patient received infliximab 5 mg/kg at standard time intervals on weeks 0 and 2, which resulted in normalisation of bowel movements. Due to concomitant progression of malignant disease, treatment was not continued. The patient was considered for third line systemic oncological therapy, but his general health deteriorated and he died 6 months later due to septic pneumonia.

### Case 2

**FIGURE 2.**
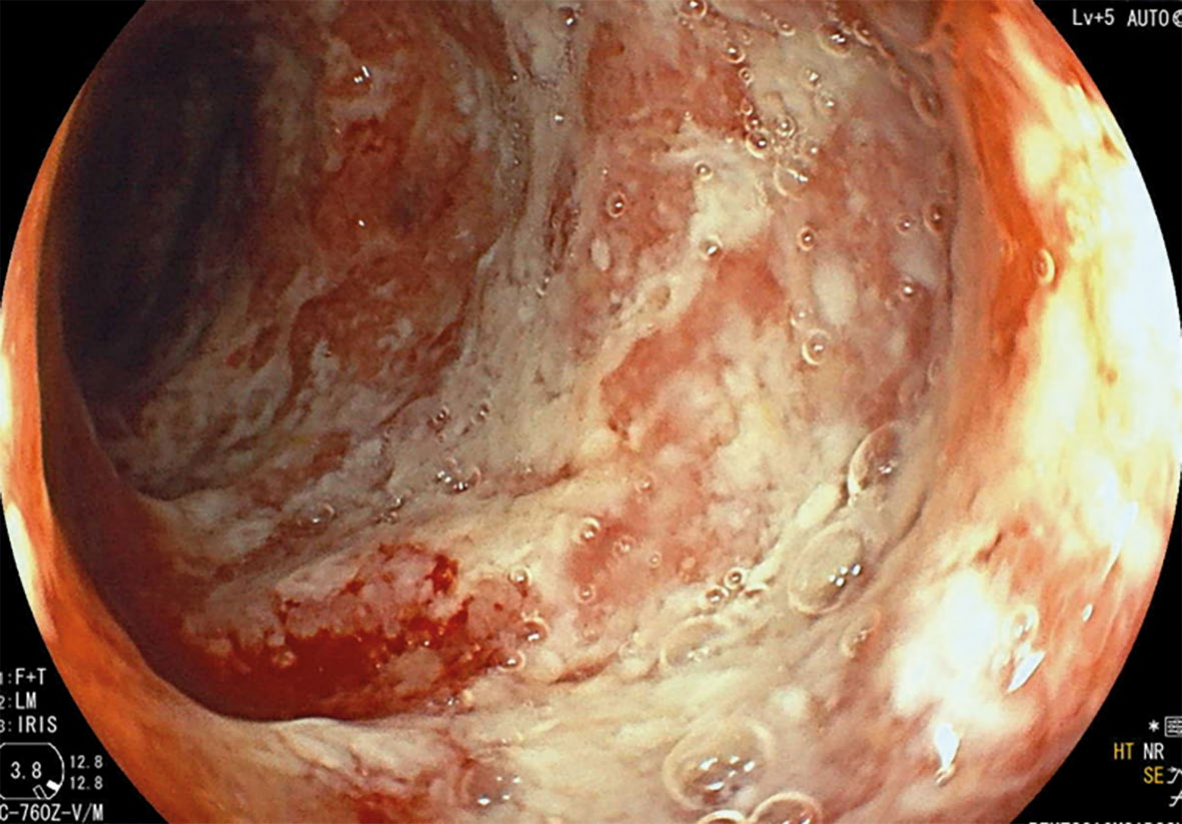
Endoscopic finding in case 2.

A 75-year-old man with primary metastatic lung adenocarcinoma received the PD-1 inhibitor pembrolizumab as first-line systemic oncologic treatment. After 2 applications of pembrolizumab, he developed frequent discharges of small amounts of mucus. Immunotherapy was stopped, stool cultures were performed and were negative. Despite supportive symptomatic treatment and loperamide, the condition worsened and due to diarrhoea grade 3, systemic corticosteroid methylprednisolone was started at a dose of 1 mg/kg/day orally. After normalisation of bowel movements, the dose of methylprednisolone was tapered and discontinued after 6 weeks. 5 days after discontinuation, mucous discharge recurred up to 5 times daily and abdominal pain appeared, with a CRP of 157 mg/L, mild leucocytosis (10.27x10^9^), and normal procalcitonin levels (0.11 mg/L). Emergency imaging (plain abdominal X-ray, chest X-ray, and abdominal CT) excluded life-threatening complications, and signs of pancolitis were described. The patient was admitted to hospital where, after serological investigations (viral hepatitis, HIV and Quantiferon test), he received methylprednisolone at a dose of 2 mg/kg/day intravenously. Endoscopic examination showed diffuse mucosal inflammation with ulcerations, endoscopic Mayo score 3 (Figure 2).

Histology confirmed marked active colitis with ulceration, architectural changes of the crypts, with extensive areas of acute cryptitis and crypt abscesses. Decision of multidisciplinary team was to treat the patient with infliximab at a dose of 5 mg/kg at standard time intervals (weeks 0, 2 and 6). After only one dose of infliximab, CRP dropped to 16 mg/L and after the 2^nd^ dose to normal levels, the bowel movements normalised. After the 3^rd^ dose of infliximab, the treatment was stopped and the patient is being further managed by the treating oncologist.

### Case 3

**FIGURE 3.**
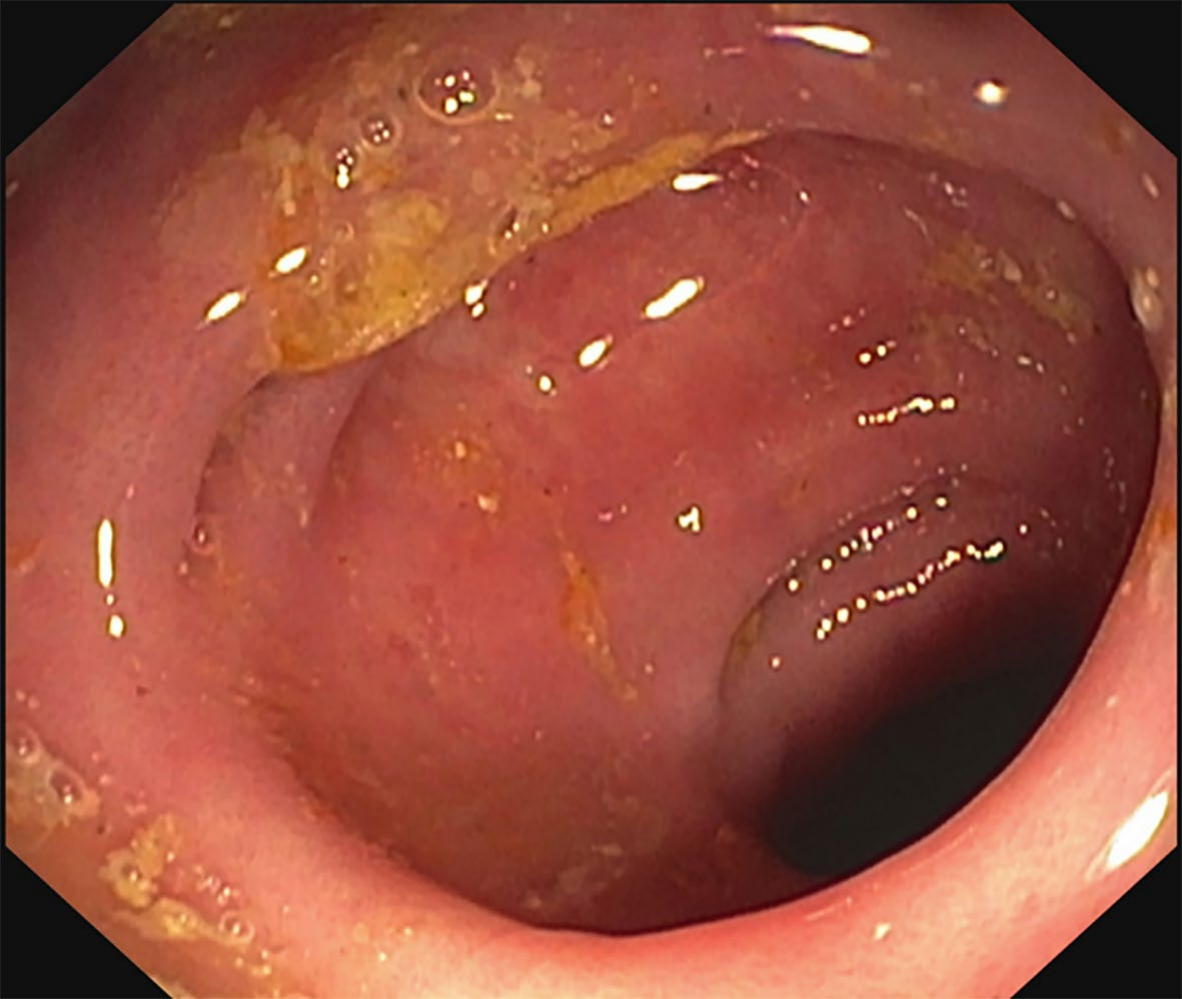
Endoscopic finding in case 3.

A 72-year-old man received the PD-1 inhibitor nivolumab at 14-day intervals as second-line systemic oncologic treatment for metastatic renal cell carcinoma. After the first month of treatment, diarrhoea, initially grade 2, started. Due to the escalation of diarrhoea to grade 3 despite immunotherapy withdrawal and supportive treatment (dietary measures, oral rehydration, loperamide), methylprednisolone was introduced at a dose of 1 mg/kg/day, to which he responded well. The dose of methylprednisolone was gradually tapered and discontinued. 10 days after discontinuation, diarrhoea recurred and he was restarted on corticosteroid therapy and referred for colonoscopy. Endoscopy showed erythema of the sigmoid mucosa and hyperaemia of the rectal mucosa, endoscopic Mayo score 1 (Figure 3).

Histology showed infiltrates of plasma cells in the lamina propria, few lymphocytes, eosinophilic and neutrophilic granulocytes with minimal signs of cryptitis, without crypt micro-abscesses. Although histological picture was non-specific, the pathologist considered it to be consistent with ICPI-induced colitis. Following the decision of the multidisciplinary team, the patient was treated with vedolizumab at a standard dose of 300 mg intravenously at time intervals week 0, 2, 6 and 14. CT of the chest and abdomen showed stagnation of malignant disease and no signs of colitis complications. The patient continues follow-up by the treating oncologist.

## Conclusions

Immunotherapy represents a breakthrough in treatment of various cancers, but can trigger immune-mediated side effects, of which ICPI-induced colitis is most common. In most cases, the course of colitis is mild or may be effectively treated with corticosteroid therapy. Small proportion of patients require treatment with biologic agents, and rarely, more severe form of colitis may trigger life-threatening complications. In these cases, cooperation between oncologist and gastroenterologist is vital to establish a rapid diagnosis with endoscopic evaluation and timely escalation of treatment.
